# Oral administration of inosine produces antidepressant-like effects in mice

**DOI:** 10.1038/srep04199

**Published:** 2014-02-26

**Authors:** Junko Muto, Hosung Lee, Hyunjin Lee, Akemi Uwaya, Jonghyuk Park, Sanae Nakajima, Kazufumi Nagata, Makoto Ohno, Ikuroh Ohsawa, Toshio Mikami

**Affiliations:** 1 Graduate School of Health and Sport Science, Nippon Sport Science University, Tokyo, Japan; 2 Department of Health and Sports Science, Nippon Medical School, Kawasaki, Japan; 3 Department of Biochemistry and Cell Biology, Institute of Development and Aging Science, Graduate School of Medicine, Nippon Medical School, Kawasaki, Japan; 4 Kyoritsu Women's Junior College, Tokyo, Japan; 5 Biological Process of Aging, Tokyo Metropolitan Institute of Gerontology, Tokyo, Japan

## Abstract

Inosine, a breakdown product of adenosine, has recently been shown to exert immunomodulatory and neuroprotective effects. We show here that the oral administration of inosine has antidepressant-like effects in two animal models. Inosine significantly enhanced neurite outgrowth and viability of primary cultured neocortical neurons, which was suppressed by adenosine A_1_ and A_2A_ receptor agonists. Oral administration of inosine to mice transiently increased its concentration in the brain and enhanced neuronal proliferation in the dentate gyrus, accompanied by phosphorylation of mitogen-activated protein kinase and increase in transcript level of brain-derived neurotrophic factor. In stress models, oral inosine prevented an increase in immobility time in forced swim test after chronically unexpected stress and mitigated a reduction in sucrose preference after chronic social defeat stress. These results indicate that oral administration of inosine has the potential to prevent depressive disorder via adenosine receptors.

A purine nucleoside, inosine, is commonly found in meat and fish. Adenosine deaminase converts adenosine to inosine. Unlike adenosine, little attention has been paid to the physiological roles of inosine. However, recent studies demonstrate that inosine has neuroprotective, cardioprotective and immunomodulatory effects. Inosine increases neuronal survival and neurite outgrowth *in vitro*, enhances axon regeneration and axonal sprouting after damage of the central nervous system, and promotes recovery in rodent models of stroke and focal brain trauma^1^,^2^,^3^. Administration of inosine improves myocardial function during acute left ventricular failure and protects myocardial and endothelial function after heart transplantation^4^,^5^. Inosine also augments mast-cell degradation, suppresses macrophage, lymphocyte and neutrophil activation, and attenuates inflammatory diseases^6^,^7^.

Extracellular inosine binds to subtypes of G-protein-coupled adenosine receptors (A_1_, A_2A_, A_2B_ and A_3_), each of which exhibits a specific tissue distribution and pharmacological profile^8^,^9^. The protective effects of inosine on primary cultured neurons were attenuated by the treatment of mice with selective A_1_ receptor antagonists^10^. A_2A_ receptor knockout mice provide evidence that some of the immunomodulatory effects of injected inosine are mediated by A_2A_ receptors^11^. Moreover, inosine increases the rates of glycogenolysis and gluconeogenesis via A_3_ receptor on hepatocytes^12^. The binding of inosine to the receptors activates intracellular signaling responses including the mitogen-activated protein kinase (MAPK) pathway, which is essential for inosine-mediated neuroprotection^13^.

A number of studies such as cohort surveillance of dietary patterns and depressive symptoms and animal studies of vitamins, minerals, fatty acids and polyphenolic components (catechins, curcumin, and resveratrol) have shown that nutrients are related to the prevention of depressive conditions^14^,^15^,^16^. It has been proposed that hippocampal neurogenesis might play an important role in mood disorder^17^. Indeed, hippocampal neurogenesis is reduced by stressful experience and decreases in animal models of depression^18^. Many treatments including electroconvulsive therapy and common antidepressant drugs for depression have been shown to enhance neurogenesis^19^,^20^. Changes in diet are also likely to influence neurogenesis. Calorie restriction enhances adult hippocampal neurogenesis in mice, a process most likely regulated via brain-derived neurotrophic factor (BDNF)^18^.

Because inosine is a common component of food and has potential neuroprotective function, we hypothesized that the oral administration of inosine is beneficial for the prevention of psychiatric diseases. Actually, intraperitoneal administration of adenosine produces an antidepressant-like effect in mice via A_1_ and A_2A_ receptors^21^, and activation of A_1_ receptor induces an antidepressant-like effect in rats^22^. We show here that oral administration of inosine has an antidepressant-like effect in mice, suggesting that inosine has potential as a brain food for preventing depressive disorders.

## Results

### Inosine improves cell viability in cultured neocortical neurons

**Figure 1:**
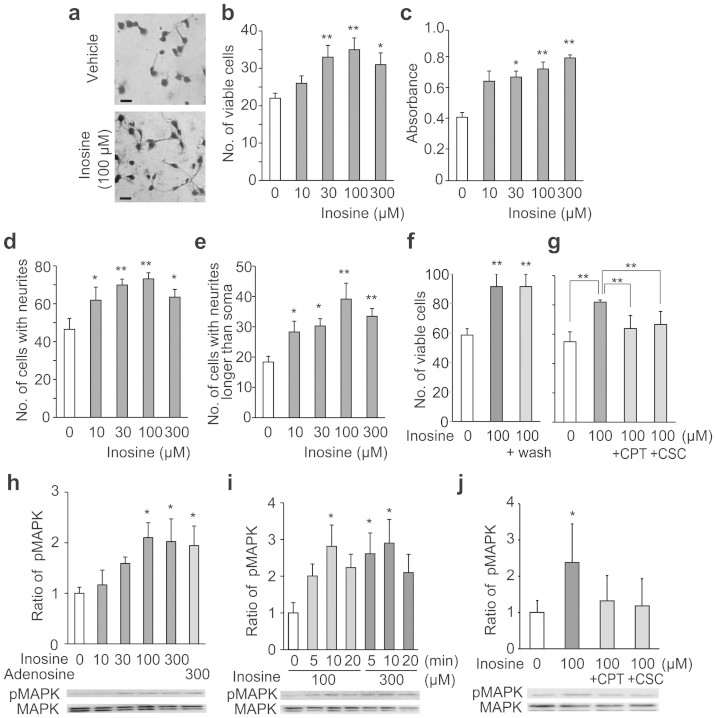
Inosine promotes neuronal viability and neurite outgrowth. Neocortical neurons were incubated with inosine for one day. (a) Surviving neurons stained with CBB. Scale bar: 20 μm. (b, c) Dose-dependent effect of inosine on cell viability. Surviving neurons were counted under a microscope (n = 5) (b). Viability was measured by a colorimetric method with water-soluble tetrazolium compound (n = 4) (c). (d), (e) Dose-dependent effect of inosine on neurite outgrowth. After staining with CBB, the number of cells bearing neurites (d) and that of cells extending neurites longer than the soma (e) was counted (n = 5). *P < 0.05, **P < 0.01, versus 0 μM of inosine. (f) Effect of transient treatment with inosine on neuronal viability. Two hours after the addition of 100 μM inosine, neurons were washed with fresh medium and further incubated for one day. Surviving neurons were counted (n = 6). **P < 0.01, versus 0 μM of inosine. (g) Inhibitory effects of adenosine receptor antagonists on neurotrophic function of inosine. CPT or CSC (10 μM) was added to neurons 1 hour before supplementation of 100 μM inosine. One day after the incubation, surviving neurons were counted (n = 5). **P < 0.01, versus 0 μM of inosine, inosine + CPT and inosine + CSC, respectively. (h–j) Inosine-induced phosphorylation of MAPK in neurons. Dose-dependent phosphorylation of MAPK was determined in neurons 10 min after supplementation of the indicated amount of inosine. Adenosine was used as a positive control (n = 3) (h). For time course analyses, neurons were harvested at 5, 10 and 20 min after supplementation of inosine (n = 3) (i). CPT or CSC (1 μM) was added to neocortical neurons 1 h before supplementation of 100 μM inosine, and neurons were harvested at 10 min after the supplementation (n = 5) (j). Cell extract was analyzed by Western blotting and quantitation of the density of bands representing phosphorylated MAPK (pMAPK) was assessed by densitometric scanning and expressed relative to the band at 0 μM of inosine. *P < 0.05, versus 0 μM of inosine. Data represent mean ± s.e.

Neocortical neurons isolated from Wistar embryonic rat were cultured in medium supplemented with or without inosine. After 1 day, neurons incubated with 100 μM inosine appeared to extend a larger number of neurites than those with vehicle (Figure 1a). Microscopic examination and a colorimetric method with water-soluble tetrazolium compound revealed that 30–300 μM inosine significantly improved cell viability in a dose-dependent manner compared with that in the non-supplemented medium (Figure 1b, c). Supplementation of 10–300 μM inosine significantly increased the numbers of neurons with neurites and those with neurites longer than the diameter of the soma in a dose-dependent manner (Figure 1d, e). Transient treatment with inosine for 2 h further improved cell viability (Figure 1f).

To examine whether inosine improves cell viability via adenosine receptors, 8-cyclopentyl theophylline (CPT) and 8-(3-chlorostyryl) caffeine (CSC), A_1_ and A_2A_ receptor antagonists, were added to the medium 1 h before inosine supplementation, respectively. Cell viability measured microscopically after 24 h of culture was partially suppressed by incubation with 10 μM CPT and CSC (*p* < 0.01, Figure 1g). Next, we examined the phosphorylation of MAPK in primarily cultured neocortical neurons. After 100 or 300 μM inosine was added to the culture medium, the phosphorylation of MAPK increased and reached a maximum 10 min after the treatment (Figure 1h). This occurred in a dose-dependent manner and was significantly increased at 100 and 300 μM inosine (Figure 1i). The phosphorylation of MAPK was inhibited by pretreatment both with CPT and CSC (Figure 1j).

### Brain inosine levels increased transiently after oral administration of inosine

**Figure 2:**
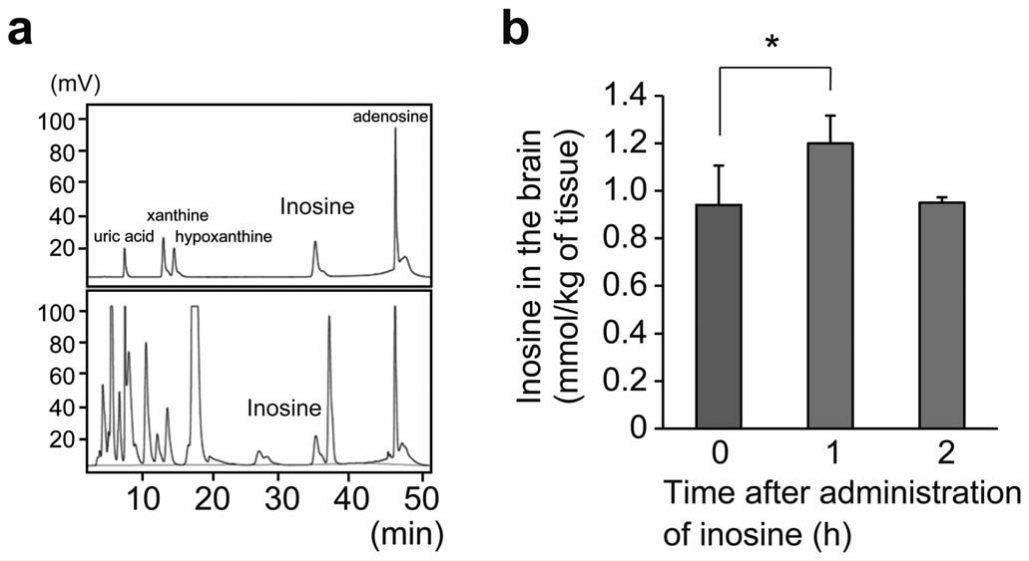
Inosine is increased in the brain after its single oral administration. (a) Typical chromatograms of HPLC. The upper image is a chromatogram of a standard mixture, and the lower one is that of the supernatant of homogenized brain in untreated mouse. (b) One hour after administration of inosine (0.33 mg/g of body weight), mice were sacrificed, and cerebral hemisphere was subjected to HPLC analysis (n = 10 per group). Data represent mean ± s.e. *P < 0.05.

To assess the possibility that orally administered inosine directly affects neuronal viability in the brain, we measured the level of brain inosine after its oral administration. Identification of an inosine peak from profiles of high-performance liquid chromatography (HPLC) was confirmed by comparison with standard mixture (Figure 2a). The amount of inosine in the brain was significantly increased 1 h after oral administration and returned to the basal level after 2 h (Figure 2b). The difference in the amount of brain inosine between before and 1 h after administration was 130 μmol/kg.

### Inosine enhances cell proliferation and transcription of BDNF in the hippocampus

**Figure 3:**
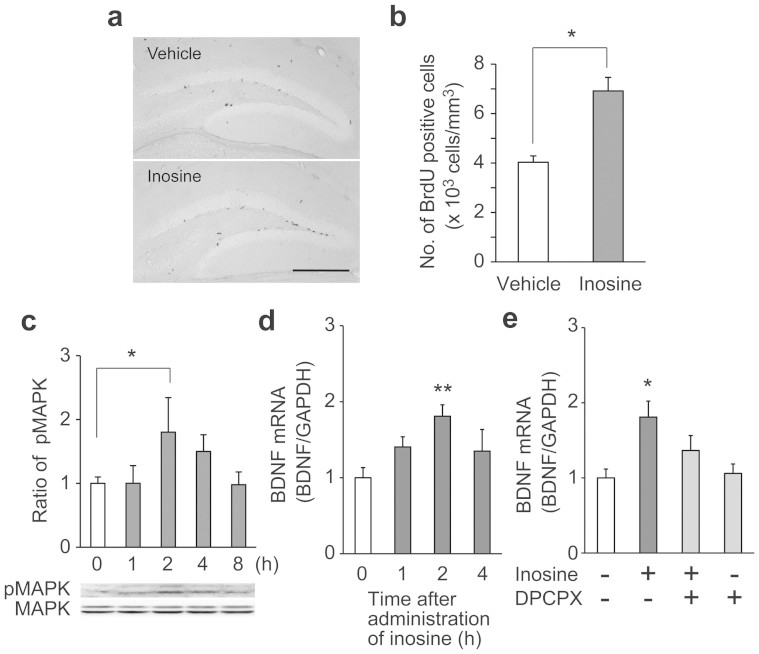
Oral single administration of inosine enhances cell proliferation, phosphorylation of MAPK and transcription of BDNF in the hippocampus. (a), (b) BrdU-positive cells were increased in the DG after administration of inosine. Mice were once injected with BrdU immediately before administration of inosine (0.33 mg/g of body weight), and sacrificed by decapitation after 24 h. Brain sections were immunostained with anti-BrdU antibody. (a) Scale bar: 100 μm. The number of BrdU-positive cells in the DG was counted (n = 9–10) (b). (c) Phosphorylation of MAPK was determined at the indicated time after oral administration of inosine (0.33 mg/g of body weight). Extract of the hippocampus was analyzed by Western blotting with anti-MAPK and phosphorylated MAPK antibodies. Quantitation of the density of bands representing phosphorylated MAPK (pMAPK) was assessed by densitometric scanning and expressed relative to the band at 0 μM of inosine (n = 5–7). (d, e) BDNF transcript levels were quantified by real-time PCR coupled with reverse transcription. Total RNA was isolated from the hippocampus. Quantitation of BDNF transcript level was expressed relative to the level at 0 μM of inosine or vehicle. Mice were sacrificed at the indicated time after oral administration of inosine (0.33 mg/g of body weight) (n = 9–10) (d). DPCPX, an antagonist of adenosine A_1_ receptor, was intraperitoneally administered to mice 30 min prior to the inosine administration, and the hippocampus was collected 2 h after inosine administration. Vehicle (n = 7), inosine with (n = 14) or without DPCPX (n = 8), and DPCPX alone (n = 8) (e). Data represent mean ± s.e. *P < 0.05, **P < 0.01.

We examined the effect of oral inosine on neuronal proliferation in the brain. The number of bromodeoxyuridine (BrdU)-positive cells in the dentate gyrus (DG) was significantly increased 24 h after oral administration of inosine compared with that upon vehicle administration (Figure 3a, b). Phosphorylated MAPK in the hippocampus also increased and reached a maximum 2 h after the administration (Figure 3c), and then gradually decreased and returned to the basal level 8 h after inosine administration.

Previous studies have reported that the activation of MAPK increases BDNF expression in the brain^16^,^23^,^24^. To investigate whether oral administration of inosine increases the expression of BDNF in the brain, BDNF mRNA in the hippocampus was quantified by real-time PCR. We found that BDNF mRNA was significantly increased 2 h after oral administration of inosine and then returned almost to the baseline (vehicle administration level) 4 h after administration. An A_1_ receptor antagonist, 8-cyclopentyl-1,3-dipropylxanthine (DPCPX), partially canceled the inosine-induced increase of BDNF mRNA (Figure 3e).

### Inosine prevents depression-like behavior in chronically stressed mice

**Figure 4:**
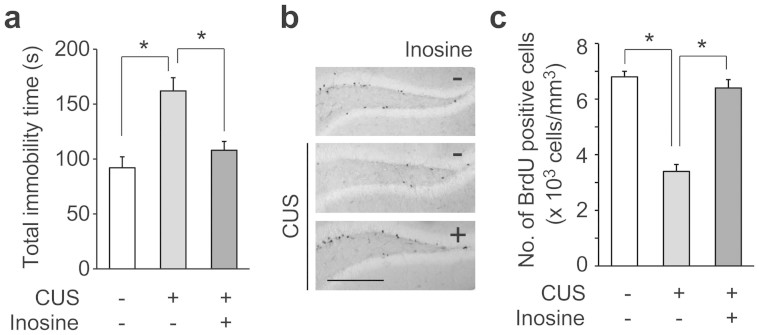
Antidepressant-like effect of inosine in CUS test. During 4 weeks of CUS, mice were allowed access to a diet supplemented with or without inosine (1 mg/g) ad libitum. (a) Inosine decreased CUS-prolonged immobility time in FST (n = 10 per group). (b), (c) Administration of inosine enhanced cell proliferation in the brain during CUS. Mice with (+) or without inosine (−) (n = 10 per group) were injected with BrdU for 3 consecutive days on the 3^rd^ week and sacrificed after the 4^th^ week of CUS. Brain sections were immunostained with anti-BrdU antibody (b). Scale bar: 100 μm. The number of BrdU-positive cells in the DG was counted (c). Data represent mean ± s.e. *P < 0.05.

After 4 weeks of chronically unexpected stress (CUS), the mice were subjected to the forced swim test (FST), which is commonly used to assess depression and hopelessness, to examine the effect of inosine on the prevention of depression-like behavior. CUS prolonged immobility time in FST, whereas the administration of inosine significantly decreased it (Figure 4a). The number of BrdU-positive cells in the DG was significantly lower in the CUS mice than in both control and CUS + inosine mice, whereas there was no significant difference between control and CUS + inosine mice (Figure 4b, c). These findings suggest the possibility that oral administration of inosine prevents CUS-induced onset of depression-like behavior with improvement of neuronal proliferation in the brain.

### Inosine prevents depression-like behavior in chronic social defeat mice

**Figure 5:**
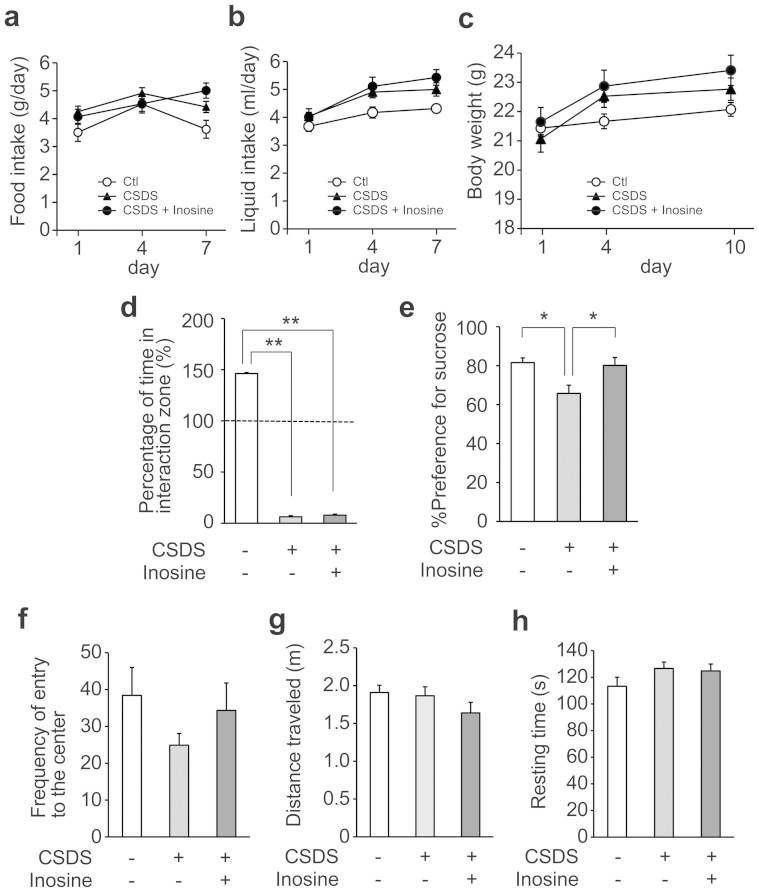
Antidepressant-like effect of inosine in CSDS test. During 10 days of CSDS, mice were allowed access to water supplemented with (n = 10) or without inosine (1 mg/ml) (n = 12) ad libitum. Control mice (Ctl; n = 12) were individually housed without stress. (a–c) Food intake, liquid intake and body weight did not show any significant difference between stressed mice with and without inosine during CSDS test. (d) Inosine did not prevent aversive responses to aggressor mice. Social interaction was assessed by the following calculation: 100 × (interaction time, aggressor present)/(interaction time, aggressor absent). (e) Inosine prevented CSDS-induced reduction of preference for sucrose. Data represent mean ± s.e. *P < 0.05, **P < 0.01. (f–h) After CSDS, anxiety-like behavior and locomotive activity were evaluated by 10 min of OFT. Frequency of entry to the center in the arena by socially defeated mice tended to decrease compared with those of control mice, whereas inosine prevented this (f). However, there were no significant differences in distance traveled (g) and resting time (h) among the three groups. Data represent mean ± s.e.

The antidepressive effect of the oral administration of inosine was examined using another depression model, chronic social defeat stress (CSDS). Mice evaluated as susceptible to depression were subjected to 10 days of social defeat with or without the intake of water containing inosine. To evaluate aversive responses to aggressor (resident) mice, all mice were subjected to social interaction test. Undefeated control mice spent most of their time interacting socially when an aggressor mouse was presented, while defeated mice both with or without inosine spent significantly less time in close proximity to the target mouse, which indicated that inosine did not prevent intense aversive responses to aggressor mice (Figure 5d). Food intake, liquid intake and body weight also showed no significant difference between stressed mice with and without inosine (Figure 5a–c). Depression-like behavior after CSDS was evaluated by the sucrose preference test, which assesses anhedonia by measuring reduction in the preference for sucrose-containing water^25^. Socially defeated mice exhibited a significantly reduced sucrose preference ratio compared with the control mice, whereas inosine prevented this (Figure 5e). Anxiety-like behavior and locomotive activity were evaluated by OFT. Frequency of entry in the central area by socially defeated mice tended to decrease compared with those of control mice (Figure 5f), whereas inosine tended to prevent this. However, there were no significant differences in distance traveled and resting time among the three groups (Figure 5g, h). These results further suggest that the oral administration of inosine contributes to the prevention of depression-like behavior.

## Discussion

To the best of our knowledge, our study shows for the first time that the oral administration of inosine prevents the onset of depression-like behavior in chronically stressed mice. It is likely that inosine in food is transported to the brain, activates adenosine receptors and functions as a neuromodulator. Previous studies using cell culture systems indicated that inosine increases cell proliferation and the growth of dendrites in PC12 cells and primary cultured cells from the cerebellum and the spinal cord^13^,^26^,^27^. We also confirmed that the addition of inosine to primary cultured neocortical neurons significantly improved cell viability and neurite outgrowth in a dose-dependent manner (Figure 1a–e). We found that transient treatment with inosine for 2 h was enough to enhance neuronal survival *in vitro* (Figure 1f). The observation supported the results in mice that the transient elevation of inosine (Figure 2) in the brain enhanced neuronal proliferation. These findings show that inosine is potentially an effective neurotrophic factor.

Inosine is reported to bind directly to A_1_, A_2A_ and A_3_ receptors. In particular, the activation of A_1_ and/or A_2A_ receptors is reported to be involved in neuromodulatory functions. Most of the sedating, anxiolytic, seizure-inhibiting and protective actions of adenosine are mediated by A_1_ receptor on the surface of neurons and glia^28^. Its activation reduces neuronal excitability in post-synaptic neurons and suppresses neurotransmitter release in the pre-synaptic neurons. On the other hand, the function of A_2A_ receptor is controversial. The neuroprotective function of purine nucleosides against hypoxia-induced cell death in PC12 cells is mediated by activation of A_2A_ receptor^29^. However, its antagonists inhibit motor deficits induced by dopamine antagonists and dopaminergic neurotoxins^30^. We observed that both A_1_ and A_2A_ receptor antagonists partially inhibited the protective effects of inosine on primary cultured neurons (Figure 1g), and A_1_ antagonist partially inhibited the inosine-induced increase in BDNF transcription in the brain (Figure 3e), indicating that both receptors could be involved in the neuroprotective and neuromodulatory functions of inosine. A previous study also indicated that antinociceptive effects of inosine in the acetic acid test were attenuated by the treatment of mice with A_1_ and A_2A_ receptor agonists^31^. We observed that the addition of inosine immediately increased the phosphorylation of MAPK (Figure 1h, i). Several findings have demonstrated that the linkage of adenosine receptors to the p42/44 MAPK pathway apparently plays a pivotal role in inosine-mediated cellular protection^13^,^26^. Inhibitory effects of adenosine receptor antagonists on phosphorylation of MAPK (Figure 1j) further supported previous findings.

The inosine level in the mouse brain was significantly increased 1 h after oral administration (Figure 2b), indicating that oral inosine may be transported into the brain. Because the inosine plasma concentration is submicromolar order^49^, it is unlikely that blood contamination affects the inosine concentration in the brain. Exogenous inosine is taken up rapidly in intestinal epithelial and liver cells, and mostly metabolized to hypoxanthine^32^, while unchanged inosine is speculated to be transported into the brain. Studies using radioactive tracers suggest that inosine crosses the blood-brain barrier efficiently^33^. Transporters specific to inosine in the blood-brain barrier have been identified as hENT1, hENT2, hCNT2, and hCNT3^34^. There is a certain intrinsic level of brain inosine that results from adenosine degradation. However, HPLC analysis revealed that the increase in brain inosine 1 hr after administration was 130 μmol/kg, close to the effective concentration for cell viability in the primary culture (Figure 1), indicating that the transient elevation of inosine is sufficient to modulate neuronal function.

We found that single oral administration of inosine to untreated mice increased neuronal proliferation in the DG (Figure 3a, b). It was previously shown that the activation of A_1_ receptor induces neural stem cell proliferation^35^. Adult hippocampal neurogenesis has been linked directly to cognition and mood. For example, memantine, which is used clinically for the treatment of Alzheimer's disease, promotes cell proliferation in the DG^36^. A direct role of neurogenesis in depression-like behavior was observed in several situations that are used to assess antidepressant efficacy and characterize the development of depression-like phenotypes in response to chronic stress. Indeed, the administration of adenosine either intraperitoneal or intracerebroventricular showed an antidepressant-like effect in mice via A_1_ and A_2A_ receptors in FST and tail suspension test^21^. Our results of FST after CUS revealed that the oral administration of inosine shortened the longer stress-induced immobility time, an index of behavioral despair, indicating that inosine could produce an antidepressant-like effect (Figure 4a). As expected, feeding with inosine significantly improved the stress-induced decrease in BrdU-positive cells in the DG (Figure 4b, c). We found that 2 h after administration of inosine, both phosphorylation of MAPK and transcription of BNDF significantly and simultaneously increased in the brain (Figure 3c, d). Since a number of studies have reported that BDNF transcription increases after the enhancement of MAPK phosphorylation^16^,^23^, it is likely that increase in MAPK phosphorylation by uptake of inosine promotes transcription of BDNF in the brain. Neurotrophic factors such as BDNF are implicated in the control of cell proliferation and survival. The observed increase in BDNF transcription was partially inhibited by A_1_ receptor antagonist (Figure 3e), indicating that *in vivo* neuromodulatory effects of inosine are related to activation of the MAPK cascade via adenosine receptors, similar to its *in vitro* neuroprotective activity.

We examined the antidepressant-like effect of inosine with two models of depression, CUS and CSDS. After CUS, a pathogenic factor that leads to a mixed anxiety/depression state, FST revealed that the administration of inosine shortened the immobility time of the stressed mice (Figure 4a). Feeding with inosine significantly improved the number of BrdU-positive cells in comparison with that of the stressed mice (Figure 4b, c). Because of BrdU injection in three consecutive days, the number of BrdU-positive cells was higher than that of the mice given single administration of inosine (Figure 3b). The mice subjected to CSDS, which is closer to stress-linked human conditions^37^, totally avoided an aggressor with or without inosine in the social interaction test, and the stress was confirmed to be given equally to each mouse (Figure 5d). Meanwhile, in the sucrose preference test, which evaluates depressive status by measuring anhedonia, it was shown that oral administration of inosine promoted low intake of sucrose in the defeated mice (Figure 5e). Although CSDS has been shown to induce body weight gain in several studies^38^,^39^, we did not observe any significant increases in body weight in the stressed mice with or without inosine (Figure 5c). It appears that oral administration of inosine might have no effect on overweight in CSDS; however, it prevents anhedonia, a brain-derived behavior representative of depression. The decline in sucrose intake in mice is consistent with the loss of interest in pleasurable activities that is a classic symptom of clinical depression^48^. Taken together, these results show that the oral administration of inosine produces an antidepressant-like effect in mice, indicating that uptake of inosine can be effective on human depression.

Inosine is a common component of many foods and a derivative of adenosine produced in the processes by which energy is produced, with little aversive effects. Inosine has so far been studied by intraventricular or intraperitoneal injection, but we demonstrated that the oral administration of inosine could function as a method to provide brain food. Further studies including the interaction between inosine and antidepressants are needed; however, in treating depressive disorders, inosine has the potential to be an innovative remedy in the future.

## Methods

### Animals

Animals were purchased from Sankyo Lab, Japan. Wistar embryonic rats were used for the primary culture of neocortical neurons. Male ICR mice were purchased for treatment with the protocol for CUS and the determination of levels of inosine, phosphorylated MAPK, BDNF mRNA and cell proliferation in the brain. Male C57BL/6J mice and male ICR mice were purchased for treatment with the protocol for CSDS as intruder and resident (aggressor) mice, respectively. The care and use of laboratory animals were in accordance with the National Institutes of Health guidelines. This study was approved by the Animal Care and Use Committee of Nippon Medical School.

For single oral administration of inosine (Sigma), it was dissolved in tap water and given with a feeding needle at a dosage of 330 mg/kg body weight. During CUS and CSDS, mice were allowed access to an inosine-supplemented diet (1 mg/g) and inosine-supplemented tap water (1 mg/ml) ad libitum, respectively. At that time, the mice were given inosine at a daily dosage of about 200 mg/kg body weight.

### Primary culture of neocortical neurons

Neocortical neurons were prepared from 16.5-day old Wistar rat embryos as described previously^40^ with slight modifications. In brief, after mechanical dissociation of neocortical tissues, cells were resuspended in minimal essential medium (MEM, Invitrogen) supplemented with 1% fetal bovine serum, 1% glutamine, 0.1% putrescine and penicillin-streptomycin, and then put onto poly-l-lysine-coated plates at a density of 5 × 10^6^ cells/cm^2^. After 3 h of incubation, the medium was changed to MEM supplemented with 10 μg/ml insulin, 5.5 μg/ml transferring and 6.7 ng/ml sodium selenite. Neuronal identity was confirmed by immunostaining with anti-TuJ1 (Sigma) and anti-glial fibrillary acidic protein (Merck Millipore). Preparations containing 95% neurons were used for the experiments.

To examine the effect of inosine on cell viability, cultured medium was changed to MEM with or without inosine, and cells were incubated for 1 day. Surviving cells were stained with Coomassie Brilliant Blue (CBB; Sigma) and counted in four randomly selected fields under a microscope with 40 × magnification. The number of cells with neurites longer than the diameter of their soma was also counted. Cell viability was further determined using a water-soluble tetrazolium compound (Nacalai, Japan) according to the manufacturer's instructions.

To examine the effect of adenosine receptor antagonists *in vitro*, 1 hour after the incubation of cells with CPT (Sigma) or CSC (Wako, Japan) at a final concentration of 1 μM, the medium was changed to inosine-containing MEM and the cells were incubated for 1 day.

### CUS

CUS was conducted according to a modified version of the method described previously^41^. Stressed mice were housed individually in a small 10 × 10 × 10 cm compartment of a multicompartment cage for the remaining time to avoid aggression, and exposed to two stressors daily, in the morning and at night. The stressors used in this study were immobilization, cage tilting, wet bedding, rat's odor, cage rotating, swimming in water (18°C), storage in a cold room, lights on overnight, crowding overnight, food/water deprivation overnight and stroboscope. During CUS, mice were fed a diet supplemented with or without inosine. Unstressed mice were housed in standard-sized cages each containing 5 mice, and handled daily without stress.

After 4-week of CUS, all mice were subjected to FST^42^. Mice were individually placed in a cylinder (height, 20 cm; diameter, 15 cm) filled with water (25°C), and forced to swim under conditions in which they could not escape. Movements of the mice were recorded for 6 min using a video camera. Immobility time, during which the mouse made the minimal amount of movement required to stay afloat, was determined for the latter 4 min to evaluate depression-like behavior.

### CSDS

CSDS was applied according to published protocols with slight modifications^43^. During 10 days of social defeat, intruder mice were allowed to enter the living area of resident mice and interact with them for 10 min, during which they were attacked by the resident mice and displayed subordinate posturing. After 10 min of physical interaction, residents and intruders were maintained in sensory and olfactory contact for 24 h using a perforated aluminum partition dividing the resident home cage into two halves. The intruder mice were exposed to a new resident every day. Control animals were individually housed in a standard cage divided into four, and were handled daily without stress. After 10 days of defeat stress, the intruder mice were subjected to social interaction test, sucrose preference test and OFT as described below.

The social interaction test comprised two trials for 150 sec each. For the first trial, a mouse was placed into an open-field box containing a vacant wire cage. In the second trial, the vacant cage was exchanged for another cage holding an awake aggressor mouse. We measured the time spent in the interaction zone, a square surrounding the wire cage, with and without the aggressor mouse. The interaction ratio was calculated as follows: 100 × (interaction time, aggressor present)/(interaction time, aggressor absent).

For sucrose preference testing, all mice were acclimatized to two-bottle-equipped conditions for 3 days in their home cages, with a free choice between water and 1% sucrose solution being provided to each mouse^44^. The positions of the bottles were counterbalanced across the left and right sides of the testing cages and interchanged daily. Water and sucrose intakes were measured during a 12-h dark cycle by weighing the bottles before and after the test. Preference tests were performed on 2 consecutive days. Sucrose preference was calculated as a percentage as follows: [100 × volume of sucrose consumed/total volume (water and sucrose) consumed].

The OFT was performed to measure locomotor activity using the open field of a white Plexiglas chamber (40 × 40 × 45 cm)^45^. Each mouse was individually placed in the left corner of an open field and locomotion was recorded for 10 min. Entry into the center, distance traveled and resting time were estimated by analyzing the video using Smart Junior ver.1.0.06 (Panlab).

### Immunoblot analysis of MAPK

Cells and tissues were lysed with the lysis buffer (50 mM Tris-HCl pH7.4, 1% NP-40, 0.25% sodium deoxycholate, 150 mM NaCl, 1 mM EDTA, 1 mM phenylmethylsulfonyl fluoride, 1 mM sodium orthovanadate, 1 mM sodium fluoride, 10% protease inhibitor cocktail). After Western blotting, membranes were incubated with polyclonal anti-rabbit ERK (1:1000, Cell Signaling) or monoclonal anti-mouse phospho-ERK (1:500, Cell Signaling). Densitometric analysis was performed using Image Gauge ver4.0 (Fuji Film, Japan).

### Inosine level in the brain

Cerebral hemisphere was homogenized in 12% trichloroacetic acid and centrifuged at 15,000 rpm for 10 min. Supernatant was mixed with an equal amount of 0.5 M tri-n-octylamine, mixed and placed on ice for 10 min. After re-centrifugation at 12,000 rpm for 3 min, the obtained supernatant was filtered (pore size: 0.45 μm) and applied to HPLC equipped with an ultraviolet detector (SPD-10A, Shimazu, Japan) according to a modified version of the method described previously^46^.

### Cell proliferation in the brain

Mice were intraperitoneally injected with BrdU (Sigma) at 50 mg/kg immediately before inosine administration and sacrificed by decapitation after 24 h. To determine cell proliferation during CUS, mice were intraperitoneally injected with BrdU at 50 mg/kg per day for 3 consecutive days on the 3^rd^ week of CUS and sacrificed after the 4^th^ week of CUS. The right brain hemisphere and the hemicerebellum were removed and fixed with 4% paraformaldehyde. Coronal sections (40 mm) were cut rostrocaudally with a vibratome (Leica) and immersed in phosphate-buffered saline. The sections were incubated in 2 M HCl at 37°C for 30 min to denature the DNA and immunostained with mouse monoclonal anti-BrdU (BD Pharmingen, 1:100) by using the M.O.M. kit (Vector Laboratories) as described previously^47^.

### BDNF mRNA

Mice were sacrificed by decapitation and the hippocampus was collected after the oral administration of inosine. To investigate the effect of an A_1_ receptor antagonist, DPCPX (MP Biomedicals), at 2 mg/kg of body weight was intraperitoneally administered to mice 30 min prior to the oral administration of inosine. Total RNA isolated from the hippocampus was used for real-time reverse transcription-quantitative PCR. Expression of BDNF mRNA was normalized to glyceraldehyde 3-phosphate dehydrogenase (GAPDH) expression. Primer and probe sequences for each PCR were as follows: BDNF F primer: 5′-ACCATAAGGACGCGGACTTG-3′, R primer: 5′-GAGGCTCCAAAGGCACTTGA-3′, probe: 5′-ACACTTCCCGGGTGATGCTCAGCA-3′; GAPDH: F primer 5′-CATCACTGCCACCCAGAAGA-3′, R primer: 5′-ATGTTCTGGGCAGCC-3′, probe: 5′-TGGATGGCCCCTCTGGAAAGCTG-3′.

### Statistical analysis

Data are presented as mean ± S.E. Statistical analysis was performed using one- or two-way ANOVA. To characterize the differences between groups further, Fisher's PLSD post hoc test was used. A value of *P* < 0.05 was accepted as the level of significance.

## Author Contributions

J.M., I.O. and T.M. conceived and designed the study. J.M. and I.O. performed cell experiments. J.M., Ho.L., Hy.L., A.U., P.J., S.N. and K.N. performed other experiments. J.M., M.O., I.O. and T.M. analyzed the data and wrote the manuscript.
